# Effectiveness of Educational Programs on Nutritional Behavior in Addicts Referring to Baharan Hospital, Zahedan (Eastern of IR Iran)

**DOI:** 10.5812/ijhrba.18932

**Published:** 2014-06-10

**Authors:** Mansour Karajibani, Farzaneh Montazerifar, Alireza Dashipour, Kobra Lashkaripour, Maryam Abery, Sajedeh Salari

**Affiliations:** 1 Department of Health Promotion Research Center and Nutrition, School of Medicine, Zahedan University of Medical Sciences, Zahedan, IR Iran; 2 Department of Health Promotion Research Center and Nutrition, Pregnancy Health Research Center, School of Medicine, Zahedan University of Medical Sciences, Zahedan, IR Iran; 3 Department of Nutrition and Food Science, School of Medicine, Zahedan University of Medical Sciences, Zahedan, IR Iran; 4 Department of Psychiatry and Psychology, Baharan Psychiatric Hospital, Zahedan University of Medical Sciences, Zahedan, IR Iran; 5 Department of Nutrition, School of Medicine, Zahedan University of Medical Sciences, Zahedan, IR Iran

**Keywords:** Nutritional Status, Education, Drug Users, Iran

## Abstract

**Background:**

There are many factors which affect nutritional status of addicted such as lack o f knowledge, incorrect attitude toward modification of food pattern, and careless to food intake.

**Objectives:**

The aim of this study was to determine the effectiveness of educational program on nutritional behavior in addicts referring to Baharan hospital in Zahedan.

**Patients and Methods:**

Thirty-six addict patients were selected randomly. After recording general demographic data of patients, nutritional behaviors were determined. To determine the effectiveness of nutritional educational program, pre and post-tests were performed. Evaluation of nutritional behavior was determined as poor, fair and satisfactory levels. Statically analysis was performed by SPSS software.

**Results:**

Most addict patients had a medium level of education. Improvement in knowledge, attitude and practice (KAP) of patients after intervention was observed as follows; decreasing KAP in poor level (2.8% vs. 30.6%), (3% vs. 50%), (25% vs. 80.6%), respectively; also, increasing KAP in fair level (7% vs. 55.6 %), (15% vs. 15%), (19% vs. 7%), respectively and increasing KAP in satisfactory levels (77.8% vs. 13.8%), (50% vs. 8.3%), and (22.2% vs. 0%), respectively (P < 0.0001). There was a significant difference regarding the grade of KAP in patients based on gender, marital status, and education level after education (P < 0.0001).

**Conclusions:**

This study showed that nutritional KAP was improved in addicts. After intervention, there was a significant difference in the score of knowledge, attitude, and practice scores in patients in the current study. KAP was improved in patients after intervention including; decreased KAP in poor level and increased KAP in fair and satisfactory levels. This finding indicates that addict patients would like to modify their life style.

## 1. Background

Drug abuse and drug dependence are of serious concerns of society. Since these have negative effects on development of communities, they are considered as concerned social topics (1). According to the report of the World Health Organization, there were 200 million of drug addicts in the world in 2005. Besides, the highest addiction rate was reported in Iran with the highest prevalence of abuse observed in the age group of 20-35 (2). Drug abuse is the result of interaction between the individual, substance, and environment. On the other hand, knowledge and attitude of individuals regarding drugs and drug effect is effective in drug abuse (1). There are many factors which affect nutritional status of addicted including; lack of knowledge about negative effects of substances on bioavailability of nutrients, incorrect attitude toward modification of food pattern, careless to food intake, low levels of self-confidence and lack of motivation and physical activity. The logical action theory emphasizes the association between beliefs, attitudes, and behaviors. To modify knowledge and attitude toward the behavior should primarily be modified.

Regarding nutritional status and food security of drug users and non-drug users, all anthropometric measurements were significantly lower for drug users except for height. Besides, drug users generally maintain poorer nutritional status than non-drug users. Drug users were more prone to food insecure and to expose increasingly severe food sufficiency problems (3). Evaluation of knowledge, attitude and practice (KAP) shows important information on health and effective factors. These evaluations can be applied for determination of social health status and important factors in this regard providing sufficient health care services. Determination of nutritional knowledge, attitude and practice of patients can be useful in guiding interventional strategies for health care (4, 5).

People have difficulty controlling their intake of certain foods. Promotion of nutritional status depends on many factors. It is shown that increased knowledge can modify consumption of foods. Drug users often show signs of malnutrition due to loss of appetite and nutrient deficiencies induced by drug (6). Many studies showed an association between drug addiction and education, socio-economic status, and body mass index (BMI) (6, 7). An important factor in developing countries is poor knowledge about how the disease is spread and how it can be prohibited (8). Forbidden drug use is a well-known risk factor for food insecurity and improper nutritional status. Drug addiction changes food consumption patterns, as eating fewer meals (3) or often omission of some meals for an entire day (9) and to depend on food availability (10).

It is important to determine the mechanisms which affect food intake, mental health and behavioral factors in the association between nutritional status and health of addict patients. Public health organizations should prospectively evaluate the possible role of food supplementation and socio-structural supports on survival and treatment of addict patients. This study was designed to empower, encourage and help addict patients to apply nutritional knowledge to promote their nutritional behavior referring to addiction treatment clinic center of Baharan Hospital in Zahedan, Iran.

## 2. Objectives

The aim of this study was to determine the effectiveness of educational program on nutritional behavior in addicts referring to Baharan Hospital in Zahedan.

## 3. Patients and Methods

### 3.1. Subjects

The subjects were 40 addict patients aged 35.7 ± 9.9 years admitted to the addiction treatment Clinic of Baharan Hospital in Zahedan University of Medical Sciences (ZAUMS), Iran. Forty addict patients were first selected; four patients were excluded because of lack of cooperation and psychological and emotional problems. The rest 36 patients completed the study without any problem. All patients referred to the Center of Drug Dependency Treatment of Zahedan were informed of the study and asked for their consent to participate. They were multidrug users of substances such as heroin, opiates, crack, and morphine, and they sought detoxiﬁcation in the Central Drug Addiction Treatment Hospital (CDAT) of Baharan Hospital, Zahedan, IR Iran. Drug dependency is a disease which requires thoughtful medical and personal attention. Treatment is designed to meet patients' medical, psychological, and social needs to achieve success. The essential first step of recovery process is detoxification from drug addiction. Drug detox is the process of safely removing harmful toxins from the body caused by drug addiction and drug abuse. Body would experience withdrawal symptoms once you discontinue drug use. These symptoms can be severe and potentially life threatening and may include physical and emotional illness, trauma and discomfort, which need to be medically monitored by a professional detox program facility.

### 3.2. Methodology

The average age of addict patients was 35.6 ± 9.9 years. Demographic characteristics including age, gender, job, family size, education level, and marital status were recorded. Body weight and barefoot height were measured using digital scales (Seca, Germany) and a non-stretch tape ﬁxed to a ﬂat vertical wall. BMI was calculated by dividing body weight (kg) by the square of the height (m²), and its percentile position in the BMI indicator was determined. The questionnaire comprised four sections as

1. demographic characteristic and nutritional,
2. knowledge,
3. attitude and
4. practice. The first section contained questions regarding age, gender, job, family size, education level, and marital status.

Each section of “knowledge”, “attitude” and “practice” included 20, 10, and 10 questions. Nutritional knowledge was assessed using 3-item multiple-choice questions and the choices were "true", "false" and "I do not know". For each correct answer, 2 points, for each false answer zero, and for "I do not know" 1 point were considered. In this way, the maximum and minimum scores were 40 and 0, respectively. The mean score gained in this section was calculated for each patient as three levels of satisfactory (mean score 18-20), fair (12-17), and poor knowledge (mean score less than 12).

The "nutritional attitude" was assessed using 10 Likert questions and the choices were "I agree", "I disagree", and "no comment". Scoring was as follows: I agree 2 points, I disagree 0 points, and no comments 1 point. In this way, the maximum and minimum scores were 20 and 0 respectively. The mean score gained in this section was calculated for each patient classified as three levels including; satisfactory (mean score 17-20), fair (12-16), and poor (mean score less than 12). The questionnaire of nutritional practice included 10 questions designed based on food guide pyramid and exchange list of food. The “nutritional practice” section had multiple-choice questions with 3 answer choices of "yes", "no" and "seldom". According to answers and obtained scores, three levels of satisfactory (mean score 17-20), fair (12-16), and poor (mean score less than 11) were resulted.

### 3.3. The Process of Nutritional Education

The process of educational program was performed by nutritional experts as face to face in addiction treatment clinic of Baharan Hospital in Zahedan University of Medical Sciences (ZUMS), Iran. Educational aids included booklet, pamphlet and poster. For determination of knowledge and practice levels, pre and posttest were performed before and after the intervention during 2 months. Educational program included 2 sessions in every week and 8 sessions every month. In general, the process of educational program of nutrition prolonged 16 sessions. Each session lasted 15-20 minutes, educating addicts by nutritionist experts. Briefly, every session was discussed about the following topics:

1. Definition of food groups, food guide pyramid, nutritional value of different sources of food, process and preparation of traditional foods and their nutritional value.
2. Complications of under and over nutrition, interaction of drug and food and its role in addiction treatment and health.
3. The principal of nutrition in addicts, food pattern, and introduction to food hygiene.

To determine knowledge, attitude and practice levels of subjects, pre and post-test were performed before and after the intervention.

Construct validity of the questionnaire was assessed using principal components. Reliability of the measures and internal consistency of the items were examined using Cronbach’s α as 0.76.

### 3.4. Statistical Analysis

Statically analysis was conducted using SPSS software (version 16), with ethical points for the subjects duly observed. To compare the frequency distribution of patients in the two stage before and after education for age, gender and level of education. Wilcoxon-Ranks test and t-test were used for qualitative variables and quantitative variables respectively. Wilcoxon-Ranks test was used to compare the mean scores of nutritional knowledge, attitude, and practice expressed in three levels of fair, poor, and satisfactory before and after the educational intervention. Then after the questionnaires were filled and collected, data was coded and entered the SPSS software, version 16, and analyzed. P < 0.05 was considered as the level of significant.

## 4. Results

Of 36 addict patients, 91.7% were men and 8.3 % women, age ranging from 21 to 56 years. Mean age, family size, and BMI were 35.7 ± 9.9 years, 5.5 ± 1.9 people, and 21.3 ± 3.5 kg/m², respectively (Table 1).

**Table 1.** General Characteristics of Study Group ^a,b^

	Results
**Age, y**	35.7 ± 9.9
**Family size, person**	5.5 ± 1.9
**BMI, kg/m^2^**	21.3 ±3.6

^a^ Data are presented as Mean ± SD.

^b^ 33 males and 3 females.

Regarding the education level of addict patients, five were illiterate (13.9%), 16 medium (44.4%), 10 diploma (27.8%) and 10 upper diploma (27.8%). Twenty-two (66.7%) patients were married, 10 (27.8%) single and 2 (5.6%) divorced. The mean of knowledge, attitude and practice grade were (15.1 ± 6.1 vs. 24.3 ± 7.5), (11.8 ± 3.7 vs.16.1 ± 3.2), and (9.9 ± 2.8 vs.13.6 ± 3.9) before and after the intervention, respectively (P < 0.0001) (Table 2).

**Table 2.** Mean Score of Knowledge, Attitude and Practice in Study Group ^a^

	Before	After	t-Test	P Value
**Knowledge**	15.1 ± 6.1	24.3 ± 7.5	-4.9	P < 0.0001
**Attitude**	11.8 ± 3.7	16.1 ± 3.2	-10.1	P < 0.0001
**Practice**	9.9 ± 2.8	13.6 ± 3.9	-7.1	P < 0.0001

^a^ Data are presented as Mean ± SD.

Improvement of knowledge, attitude and practice (KAP) in patients after educational program was observed as; decreasing KAP in poor level (2.8% vs. 30.6%), (3% vs. 50%), (25% vs. 80.6%) and increasing KAP in fair level (7% vs. 55.6 %), (15% vs. 15%), (19% vs. 7%) and increasing KAP in satisfactory levels (77.8% vs. 13.8%), (50% vs. 8.3%), and (22.2% vs. 0%) respectively (P < 0.0001) (Tables 3, 4 and 5).

**Table 3.** Comparison of Knowledge Level Before and After the Intervention in Study Group ^a^

Stage ^b^	Poor	Fair	Satisfactory
**Before**	11 (30.6)	20 (55.6)	5 (13.8)
**After**	1 (2.8)	7 (19.4)	28 (77.8)

^a^ Data are presented as No. (%).

^b^ P < 0.0001.

**Table 4.** Comparison of Attitude Level Before and After the Intervention in Study Group ^a^

Stage ^b^	Poor	Fair	Satisfactory
**Before**	18 (50)	15 (41.7)	3 (8.3)
**After**	3 (8.3)	15 (41.7)	18 (50)

^a^ Data are presented as No. (%).

^b^ P < 0.0001.

**Table 5.** Comparison of Practice Level Before and After the Intervention in Study Group ^a^

Stage ^b^	Poor	Fair	Satisfactory
**Before**	29 (80.6)	7 (19.4)	0
**After**	9 (25)	19 (52.8)	8 (22.2)

^a^ Data are presented as No. (%).

^b^ P < 0.0001.

Compared to before intervention, a significant difference was observed in KAP based on gender of patients which improved from 5 (13.9%) to 28 (77.8%), 3 (8.3%) to 18 (50%), and 0 (0%) to 8 (22.2%) in satisfactory levels respectively (P < 0.0001). Moreover, a significant difference in KAP based on marital status of patients was found which improved from 11 (30.6%) to 28 (77.8%), 3 (8.3%) to 18 (50%), and 0 (0%) to 8 (22.2%) in satisfactory levels respectively (P < 0.0001) (It is not shown). There was a significant difference in KAP based on educational levels of patients which improved from 5 (13.9%) to 28 (77.8%), 3 (8.3%) to 18 (50%), and 0 (0%) to 8 (22.2%) in satisfactory levels respectively (P < 0.0001) (It is not shown).

## 5. Discussion

This study showed that nutritional knowledge, attitude and practice were higher after performance of nutritional educational program in addict patients (P < 0.0001). This finding indicates that drug addicts were successful in receiving nutritional information through the nutritional pogroms. In similar study, it showed there was association between nutritional risk and treatment retention. Given impact of nutrition on health for addicted is important aspect of drug treatment (11). Most addicts have nutritional and metabolic disorders (7, 12). Nutritional status of drug-addicted patients was very poor (3).

Findings revealed satisfactory knowledge, positive attitudes and fair practice prevailed from this study. Educational interventional program was successful to improve nutritional behavior. The present study revealed that the mean score of knowledge (15.1 ± 6.1) was lower than the score obtained from after intervention (24.3 ± 7.5). This indicates higher tendency and awareness of addict patients to nutritional and health behaviors as well as self-care behaviors. In this study, as compared to before intervention a significant difference attitude of addicts (P < 0.0001) was found. They had a better nutritional attitude after intervention regarding nutritional and healthy behaviors. Generally, addict patients have many high-risk behaviors despite they usually have an unhealthy lifestyle (1). According to the results of this study, the mean of nutritional attitude of patients was better than before intervention. This indicates that patients pay attention to health and self-care nutritional behaviors, which was mentioned in awareness section. In general, drug abuse is the result of interaction between the individual, substance, and environment. Knowledge and attitude of individuals toward drugs and drug effects is effective in its abuse. Some environmental factors involved in drug abuse are cultural factors, peer attitude toward drug abuse, parents' behavior, and regulations and policies, which restrict access to drugs (1, 13). The results of the present study indicated a positive impact of nutrition education to improve addict patients' attitude, which can modify their life style. The findings also indicated that married people have better behavior and attitude compared to bachelors. The findings of this work showed that there was tendency to change behavior in addict patients regarding nutritional status. Therefore, it is possible to reduce the onset of risky behaviors. Today’s, drug abuse is considered as an undeniable problem in our society and it calls for realistic strategies to change nutritional attitudes and behaviors.

The mean of practice grade was (9.9 ± 2.8 vs.13.6 ± 3.9) including before and after intervention respectively (P < 0.0001). In this study, practice of addicts was promoted. There was a significant difference in the grade of knowledge, attitude, and practice of addict patients in different levels (poor, fair, and satisfactory) regarding gender, marital status, and education level after education (P < 0.0001). In the current study after intervention, according to demographic criteria such as gender, marital status, and education level, there was a significant difference in knowledge, attitude, and practice scores. Although, major factors in inclination to drug abuse include; familial conflicts, seeking pleasure, curiosity of adolescents, availability of drugs, peer pressure, parents' divorce, and lower levels of self-confidence (1). This study showed that drug addicts are different regarding their nutrition. Therefore, they usually are nutritionally poor. It is necessary to ensure their nutritional condition through proper diet (3, 14). Dietary choice and quality of nutrition should be evaluated in addicts. Nutrients are not consumed separately and they are a part of dietary intake; therefore, overall dietary intake should be evaluated for the assessment of nutritional status (15). Our findings indicated that patients have a higher tendency to modify their life style. Results showed that patients pay more attention to their health and nutritional behavior that further study is needed.

Our study indicated that, after education nutritional knowledge of drug addicts was significantly improved. It was reported that attitude of addicted patients toward nicotine dependence treatment improved. While the interventional programmer likely had an effect, other factors could also have played a role in this apparent change in staff attitude (16). However, it is possible that addicts are less willing to accept some habits as unhealthy behaviors regarding their own health (17). Determination of nutritional knowledge, attitude and practice of patients can be effective to guide interventional strategies for healthcare of drug addicts. Drug habit affects nutritional status. At present, many efforts are made to encourage addicts to quit drug abuse. Other lifestyle factors associated with drug abuse having significant roles in their nutritional behavior include poverty, poor eating and exercise habits, lack of concern about nutrition and health, and diets restricted by physiological conditions (1).

Efforts to quit by increasing the level of knowledge, offering health and nutritional information, giving nutritional education via addiction cessation program can increase the level of knowledge, attitude and promotion of their eating habits. This study indicated that nutritional KAP was improved in addicts. After intervention, there was a significant difference in the score of knowledge, attitude, and practice scores in patients in the current study. Improvements of KAP in patients after intervention were decreased KAP in poor level and increased KAP in fair and satisfactory levels. This finding indicates that addict patients would like to receive nutritional information through educational programs and modify their life style. However, the findings of following studies may uncover the real picture of nutritional behavior among addicts. It is recommended to use the capacity of addicts and performance of supporting and counseling programs in parallel to clinical treatment to decrease the duration of process of treatment and increase their recovery and rehabilitation.
